# Virulence capacity of different *Aspergillus* species from invasive pulmonary aspergillosis

**DOI:** 10.3389/fimmu.2023.1155184

**Published:** 2023-03-29

**Authors:** Biao Chen, Guocheng Qian, Zhiya Yang, Ning Zhang, Yufeng Jiang, Dongmei Li, Renzhe Li, Dongmei Shi

**Affiliations:** ^1^ Laboratory of Medical Mycology, Jining No. 1 People’s Hospital, Jining, Shandong, China; ^2^ Central Laboratory, Jining No.1 People’s Hospital, Jining, Shandong, China; ^3^ Georgetown University Medical Center, Department of Microbiology & Immunology, Washington, DC, United States

**Keywords:** invasive pulmonary aspergillosis, inflammation, polyphasic taxonomy, antifungal susceptibility, virulence

## Abstract

**Introduction:**

The opportunistic filamentous fungus *Aspergillus* causes invasive pulmonary aspergillosis (IPA) that often turns into a fatal infection in immunocompromised hosts. However, the virulence capacity of different *Aspergillus* species and host inflammation induced by different species in IPA are not well understood.

**Methods:**

In the present study, host inflammation, antimicrobial susceptibilities and virulence were compared among clinical *Aspergillus* strains isolated from IPA patients.

**Results:**

A total of 46 strains were isolated from 45 patients with the invasive infection, of which 35 patients were diagnosed as IPA. *Aspergillus flavus* was the dominant etiological agent appearing in 25 cases (54.3%). We found that the CRP level and leukocyte counts (elevated neutrophilic granulocytes and monocytes, and reduced lymphocytes) were significantly different in IPA patients when compared with healthy individuals (*P* < 0.05). Antifungal susceptibilities of these Aspergillus isolates from IPA showed that 91%, 31%, 14%, and 14% were resistant to Fluconazole, Micafungin, Amphotericin B and Terbinafine, respectively. The survival rate of larvae infected by *A. flavus* was lower than larvae infected by *A. niger* or *A. fumigatus* (*P* < 0.05).

**Discussion:**

*Aspergillus flavus* was the dominant clinical etiological agent. Given the prevalence of *A. flavus* in our local clinical settings, we may face greater challenges when treating IPA patients.

## Introduction

*Aspergillus* species are the most frequently reported filamentous fungi associated with human infections (1–3). The infections can be localized to the skin, nervous system, ear, eye, or can be systemically disseminated – all are referred to as invasive aspergillosis (IA) (4, 5). Invasive aspergillosis includes a family of closely-related severe life-threatening conditions occurring in patients with compromised immune systems with high fatality rates and the worst prognoses (6). Invasive pulmonary aspergillosis (IPA) is a common and severe disease of IA and diagnosis is challenging due to the non-specific nature of symptoms (7). The incidence of IPA today continues to increase with the introduction of advanced therapies involving bone marrow or solid organ transplantation to correct haematological malignancies and even in in immunocompetent patients with prolonged systemic steroids or deteriorating pulmonary functions (8, 9).

The majority of *Aspergillus* isolates reported so far can be classified into *Aspergillus* sections such as *Fumigati*, *Circumdati, Flavi, Nidulantes, Nigri, Phialosimplex* and *Terrei*. They are opportunistic pathogens in humans and animals (10–15). Given the fact that not all *Aspergillus* species possess the same biologic attributes and antifungal susceptibility patterns, identifying *Aspergillus* species at the species level is imperative for treatment initiation. Although *Aspergillus* has been studied by taxonomists for more than 200 years, challenges remain when using morphology-based methods for species identification, e.g., to discriminate among *Aspergillus* species in the *Fumigati* section (16, 17). To date, many non-morphological methods have been developed for *Aspergillus* identification by analyzing secondary metabolites, isozyme electrophoretic patterns, molecular sequencing and gene markers such as the Calmodulin (*CaM*) gene that could be appropriate identification markers for identification of *Aspergillus* (3, 5). With these molecular methods, subgenera and section of *Aspergillus* are continually being reframed and *Aspergillus* isolates at the same time are easily identified at the species level (18–22). The second advantage of molecular methods is to help with the identification of antifungal drug resistance. Drug-resistant *Aspergillus* has become a problem worldwide in recent years (23), which worsens the currently limited treatment options for invasive aspergillosis. Nevertheless, the early diagnosis of the *Aspergillus* pathogen at the species level and the rapid identification of drug resistance still fail to meet current clinical needs.

In recent years, *Galleria mellonella* (*G. mellonella*) larvae have been used as virulence study model mainly due to having the innate immune system that is structurally and functionally similar to that of mammals. It has been utilized as a host model to evaluate virulence of other fungal microbes such as *Candida albicans* (24), *Candida auris* (25), *Conidiobolus coronatus* (26), *Beauveria bassiana* (27), and *Metarhiziun* (28). When compared to other animal models, this model is cost-effective, easily operated, safe, and exempt from the usual requirements for ethical approval of vertebrate animal uses (29, 30).

In order to provide the more rational therapeutic strategies in clinical settings, we surveyed the prevalence of *Aspergillus* species in patients with IPA or other invasive fungal infections. In addition to profiling antifungal susceptibilities of these isolates, the degree of virulence and the host inflammation induced by these different strains were evaluated in the *G. mellonella* model.

## Methods and materials

### Samples and culture

A total of 46 fungal isolates were collected from 45 patients during June 2018 and April 2022 in Clinical Laboratory at Jining No. 1 People’s Hospital, Shandong (Jining, China). Fungal cultures were grown on Sabouraud Dextose Agar (SDA), yeast extract peptone dextrose agar (YPD), blood agar (BD) at 37 °C. Isolate stocks were stored in the Laboratory of Medical Mycology of Jining No. 1 People’s Hospital, Shandong, China. Among 45 patients, 35 patients were diagnosed as IPA according to diagnostic criteria of 2019 European Organization for the Research and Treatment of Cancer/Mycosis Study Group (EORTC/MSG) consensus (7). The serum samples collected from these 35 IPA patients and 38 healthy individuals were stored at -80°C. Routine blood tests for C-reactive protein (CRP) and counts of leukocyte including neutrophilic granulocytes, lymphocytes and monocytes were performed.

### DNA extraction and sequencing

Genomic DNA was extracted from mature colonies grown on SDA plates according to the fungal DNA extraction instructions from the manufacturer (OMEGA) (21). The Calmodulin (*CaM*) gene was amplified using primer pairs (F, 5’-CCGAGTACAAGGARGCCTTC, R, 5’-CCGATRGAGGTCATRACGTGG) (31). The PCR conditions were set as follows: an initial denaturation step of 5 min at 94°C followed by 35 cycles of 30 s at 94°C, 50 s at 57°C and 1 min at 72°C, and a final elongation step of 7 min at 72°C. DNA sequencing was performed using an ABI PRISM^®^ 3730XL DNA Analyser with a BigDyeTerminater Kit v. 3.1 (Invitrogen, USA) from the General Biology Company (Anhui, China). DNA sequencing was submitted to NCBI, and the accession numbers are listed in **Table S1**.

### Phylogenetic analysis

For phylogenetic reconstruction, newly generated sequences of *CaM* were initially subjected to BLAST search (BLASTn) in NCBI website (https://www.ncbi.nlm.nih.gov). Fungal species and their sequences from Visagie and Houbraken (3) were selected. The sequence alignments were conducted using MAFFT 7 (http://mafft.cbrc.jp/alignment/server/index.html), and manually edited in MEGA 7.0.21. Maximum Likelihood (ML) analysis was implemented on the CIPRES Science Gateway portal (https://www.phylo.org) using RAxML-HPC BlackBox 8.2.10. The GTR+GAMMA substitution model with 1000 bootstrap iterations was performed. Phylogenetic trees were viewed with FigTree v.1.3.1 and processed by Adobe Illustrator CS5.

### Antimicrobial susceptibility analysis

35 strains from IPA patients were tested for antimicrobial susceptibilities. Conidial suspensions were diluted in order to obtain twice the final inoculum, which ranged from 0.4 × 10^4^ to 5 × 10^4^ cfu/mL, in a medium consisting of RPMI 1640 medium buffered at pH 7.0 with 0.165 M 3-(N-morpholine) propane-sulfonic acid (MOPS) (Gibco by Life Technologies). *Candida parapsilosis* (ATCC 22019) was used as a quality control.

Amphotericin B (Med Chem. Express Company, America), Micafungin sodium (Med Chem. Express Company, America), Voriconazole (Med Chem. Express Company, America), Itraconazole (Med Chem. Express Company, America), Terbinafine (Med Chem. Express Company, America) Furconazole (Med Chem. Express Company, America) were treated as clinical formulations and prepared according to the manufacturer’s guidelines and protocol CLSI M38-A3 in order to obtain working solutions. The concentrations ranged from 0.0313 to 16 ug/mL for Amphotericin B, from 0.015 to 8 ug/mL for Micafungin sodium, from 0.0313 to 16 ug/mL for Voriconazole, from 0.0313 to 16 ug/mL for Itraconazole, from 0.001 to 0.5 ug/mL for Terbinafine and from 0.25 to 128 ug/mL for fluconazole. Drugs were added into 96-well culture plates. While columns from 1 to 10 were filled with antifungal agents of the corresponding gradient, column 12 served as a negative control and column 11 as positive control without drug.

A total of 100 μL of the two-fold diluted conidial suspensions was inoculated into each well. Plates were incubated at 37°C for 46 to 50 h, and fungal growth in each well was assessed visually. The MIC values (minimal inhibitory concentration, MIC) of Itraconazole, Voriconazole and Terbinafine were determined by the turbidity reducing 80% or greater when compared with control wells without drug presence. For Fluconazole, the allowed turbidity reduced growth by 50% were compared to the control wells as well. For Micafungin, endpoints are often less clearly determined than those observed by amphotericin B. We also use endpoints of the minimum effective concentration (MEC) to improve reproducibility of MIC results. While the MIC is defined as the lowest concentration of drug that yields no growth, the MEC is the lowest concentration of drug that results in macroscopic changes in filamentous growth to microcolonies or granular growth when compared with growth control wells (12).

### *G. mellonella* survival test and melanization

*G. mellonella* were purchased from Wax Moth Breeding, Tianjin, China and the larvae weighing around 250 mg were chosen for the infection experiment. Three strains CCJNMMM-E-0103, CCJNMMM-E-C0034 and CCJNMMM-E-C0119 from IPA patients respectively representing *A. flavi*, *A. fumigati* and *A.nigri*, were used to measure *G. mellonella* survival rates. Each 100 μL aliquot of conidia suspension at concentrations of 1×10^6^ cfu/mL, 5×10^6^ cfu/mL or 1×10^5^ cfu/mL was injected into the last left proleg of each larva. For each tested concentration of each strain, 20 larvae were included and an additional 20 larvae injected with PBS were used as control group. Each treated group of larvae were then kept in Petri dishes at 37°C and surviving larvae were counted at 12, 24, 36 and 48 h post infection or PBS treatment. The larvae were considered dead when no movement was seen by visual inspection. The melanization of infected or PBS-treated *G. mellonella* larvae (set up according to the same infection schedules as for the survival test) was then evaluated by OD405 nm as previously reported, at 6, 12, 24 and 36 h, respectively (31).

### Histopathology analysis of *G. mellonella*

The larvae infected with the strains of CCJNMMM-E-0103, CCJNMMM-E-C0034 and CCJNMMM-E-C0119 at 1×10^6^ cfu/mL, 5×10^6^ cfu/mL and 1×10^5^ cfu/mL, respectively, were collected at 12 h for histopathological examination. Larvae were euthanized in 10% formalin that preserved and fixed the tissue anatomy at the same time. After embedded in paraffin, Periodic Acid-Schiff staining (PAS) was performed to analyze fungal structures in the tissues (25).

### Ethics statement

This study was approved by Jining No. 1 People’s Hospital, Shandong, China (2020-028). We also followed the available guidelines for strengthening the reporting of the Genetic Risk Prediction Studies. Written informed consent was obtained from all participants, or in the case of minors, from legal guardians.

## Results

### Clinical strains identification and blood routine examination

**Figure 1:**
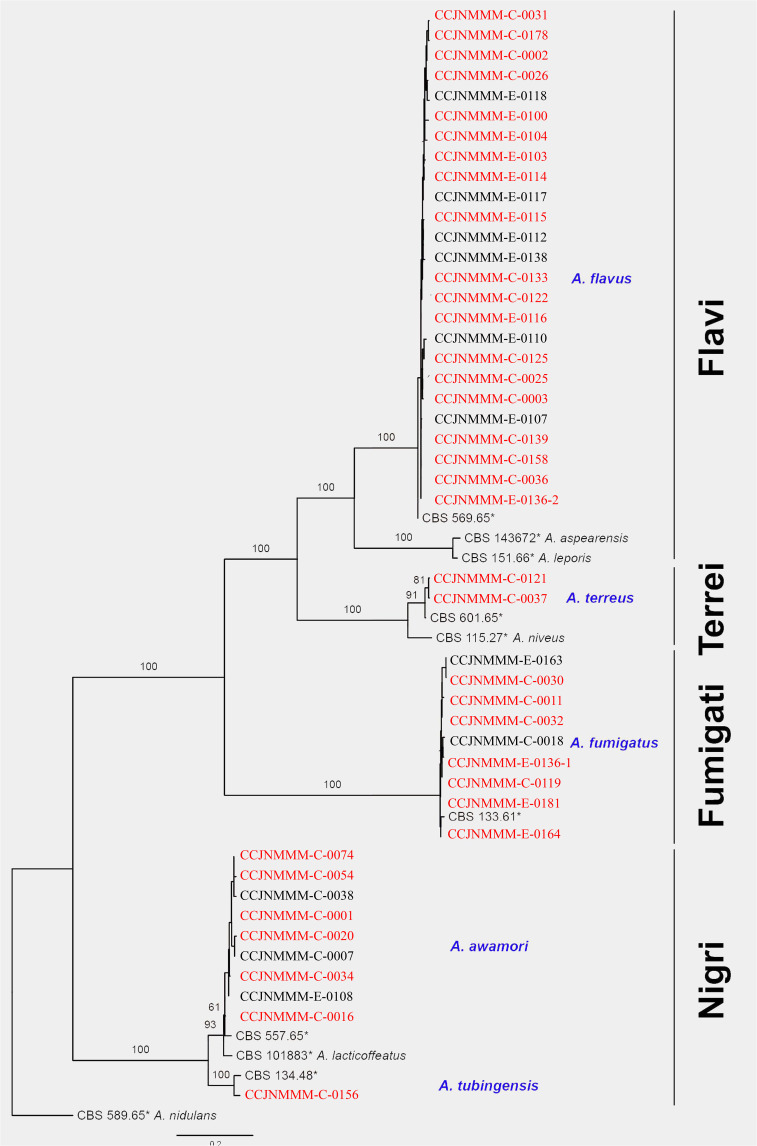
Phylogram of Diatrypaceae based on *CaM* gene sequence data. Phylogenetic analysis was performed using the maximum likelihood (ML). Bootstrapping phylogenetic trees support values above 70% are shown at the first and second positions, respectively. Thickened branches represent posterior probabilities above 0.95 from the BI (Bayesian inference). Ex-type strains are in bold, type species are denoted with the superscript “TS” and the disputable type species are denoted with the superscript “TSQ”. Strains from the current study are in blue. Red strain numbers indicated the strain from IPA.

**Figure 2:**
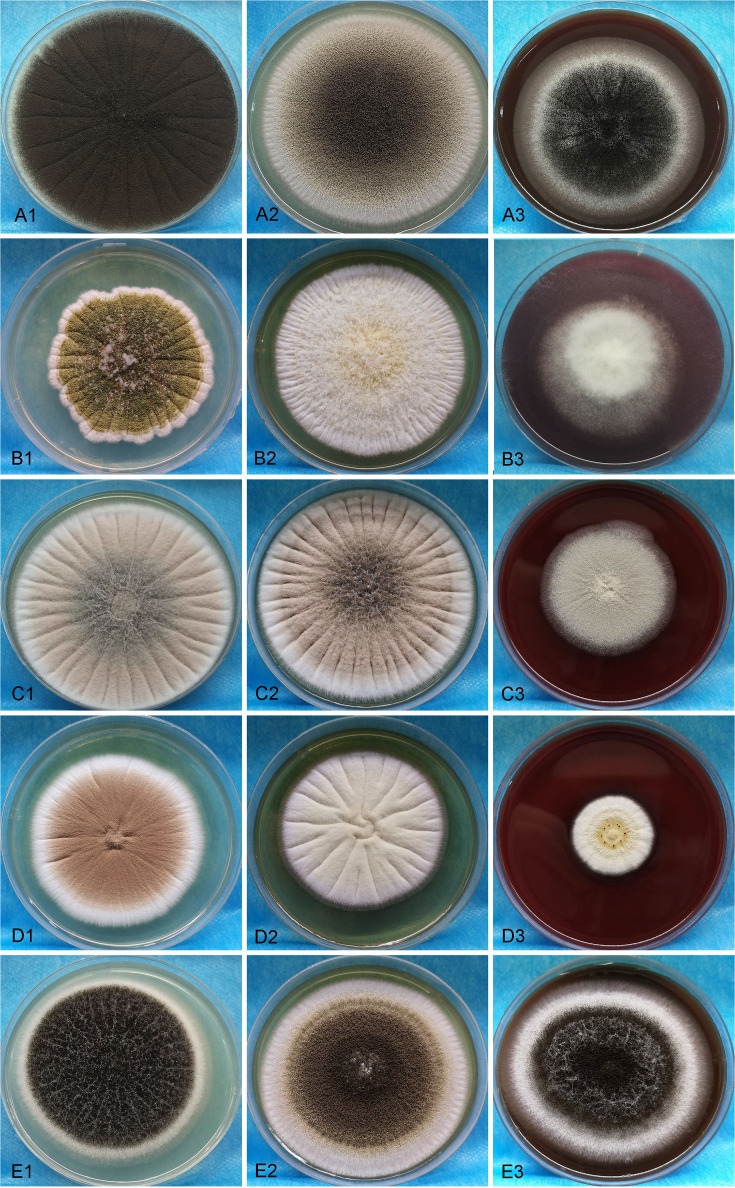
The morphologic images of clinical isolated strains were visualized on SDA, YPD, and BD plates for 5 days. **A1-A3**, **B1-B3**, **C1-C3**, **D1-D3** and **E1-E3** present *A. flavus, A. fumigatus, A. awamori, A. terreu*, and *A. tubingensis*, respectively.

**Figure 3:**
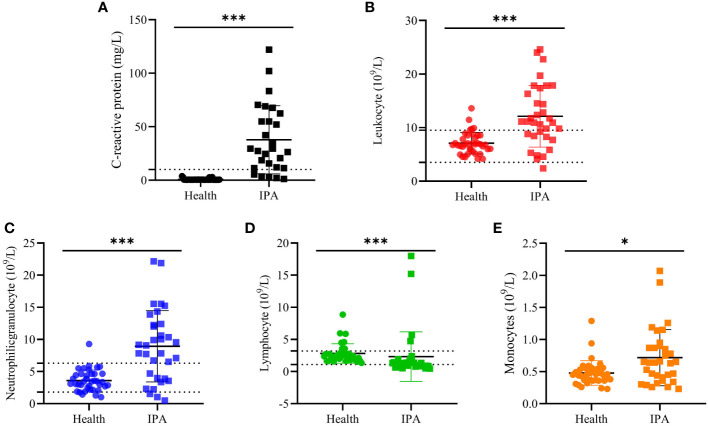
The blood routine examination. The blood routine test results from IPA and health people. **(A, C)** -reactive protein. **(B)** Leukocyte counts. **(C)** Neutrophilic granulocyte counts, **(D)** Lymphocyte counts and **(E)** monocytes count in blood samples. “*” present *p* < 0.05 and “***” present *p* < 0.001.

A total of 46 clinical *Aspergillus* isolates were obtained from 45 patients, of which 35 patients were diagnosed as IPA. Based on *CaM* gene sequencing data, these 46 isolates were separated into five distinct clades in the phylogenic tree (**Figure 1**), which includes *A. flavus* accounting for 54.3%, *A. fumigatus* (19.6%), *A. awamori* (19.6%), *A. terreus* (4.3%) and *A. tubingensis* (2.2%). The characters of each clade on SDA, YPD, and BD plates after 5 days growth are shown in **Figure 2**. The results of blood routine examination in 35 IPA patients showed that the CRP level, numbers of total leukocytes, neutrophils, lymphocytes and monocytes are significantly higher in IPA than those of health controls (*P <*0.05) (**Figure 3**).

### Antimicrobial susceptibility

The MIC ranges of 35 strains from IPA patients were determined against several currently-used antifungal drugs in clinical setting, and the results are shown in **Table 1**. The lowest MIC value for Micafungin was 0.015 μg/mL, 0.006 μg/mL for Terbinafine, 0.0125 μg/mL for Itraconazole, 0.5μg/mL for Amphotericin B, 0.125 μg/mL for Voriconazole and 8 μg/mL for Fluconazole. However, there were 91% isolates (32/35) with MIC values greater than 128 μg/mL to Fluconazole, which is followed by 31% isolates (11/35) with MIC values above 8 μg/mL to Micafungin, 14% isolates (5/35) with MIC values greater than 0.5 μg/mL to Terbinafine or 16 μg/mL to Amphotericin B, respectively.

**Table 1:** MIC values of 6 antifungal agents against *Aspergillus* strains.

Isolates		Drug (MIC: μg/mL)						
		Micafungin	Terbinafine	Amphotericin B	Voriconazole	Itraconazole		Fluoroconazole
*A.Flavi*	CCJNMMM-E-0104	>8	0.015	>16	1	0.25		>128
	CCJNMMM-E-0116	>8	0.5	>16	0.5	0.125		>128
	CCJNMMM-C-0125	>8	0.0125	>16	1	0.125		>128
	CCJNMMM-C-0036	>8	0.006	8	0.25	0.125		>128
	CCJNMMM-C-0178	0.5	0.015	4	0.5	0.5		>128
	CCJNMMM-C-0026	0.015	0.5	4	0.25	1		>128
	CCJNMMM-E-0115	1	0.015	4	0.125	0.0625		>128
	CCJNMMM-C-0025	0.25	0.015	4	0.25	0.125		>128
	CCJNMMM-C-0139	0.015	0.03125	8	0.5	0.25		>128
	CCJNMMM-C-0122	1	0.006	8	1	0.5		64
	CCJNMMM-E-0100	>8	0.015	4	0.25	0.125		>128
	CCJNMMM-E-0114	>8	0.015	4	0.25	0.0125		>128
	CCJNMMM-E-0136-2	>8	0.312	2	0.5	0.25		>128
	CCJNMMM-E-0100	>8	0.015	4	0.25	0.125		>128
	CCJNMMM-C-0002	0.25	0.015	4	1	0.25		>128
	CCJNMMM-C-0003	2	0.0625	0.5	0.5	0.5		>128
	CCJNMMM-C-0031	>8	0.015	2	0.25	0.0125		>128
	CCJNMMM-C-0158	1	0.006	>16	1	0.25		>128
	CCJNMMM-E-0103	0.125	0.006	8	0.125	0.0625		>128
*A.Terrei*	CCJNMMM-C-0121	0.0313	>0.5	4	2	0.25		>128
	CCJNMMM-C-0037	4	0.015	8	1	0.5		>128
*A.Fumigati*	CCJNMMM-E-0181	>8	0.5	>16	0.25	0.5		>128
	CCJNMMM-C-0030	0.5	>0.5	2	1	2		>128
	CCJNMMM-C-0119	0.25	>0.5	1	0.25	2		>128
	CCJNMMM-E-0136-1	>8	0.5	4	0.25	1		>128
	CCJNMMM-E-0164	0.0313	>0.5	4	0.25	0.5		8
	CCJNMMM-C-0032	2	>0.5	1	0.5	2		>128
	CCJNMMM-C-0011	2	0.125	2	1	1		>128
*A.Nigri*	CCJNMMM-C-0054	0.015	0.0625	1	1	0.5		>128
	CCJNMMM-C-0020	8	0.03125	1	1	1		>128
	CCJNMMM-C-0034	0.015	0.0625	1	1	0.5		>128
	CCJNMMM-C-0016	0.0313	0.0625	2	1	2		>128
	CCJNMMM-C-0156	0.015	0.0625	1	1	0.5		>128
	CCJNMMM-C-0074	0.015	0.0625	1	1	1		64
	CCJNMMM-C-0001	0.015	0.03125	0.5	0.25	0.25		>128

### *G. mellonella* survival tests

**Figure 4:**
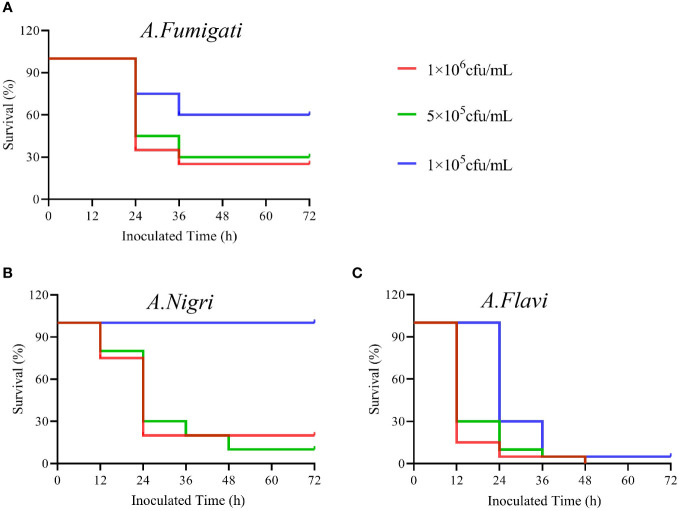
The survival rates of *G. mellonella* infected by *Aspergillus* strains. **(A)** The survival rates of *G. mellonella* infected with *A. flavi* in 1×10^6^ cfu/mL, 5×10^5^ cfu/mL and 1×10^5^ cfu/mL, respectively. **(B)** The survival rates of *G mellonella* infected with *A.nigri* in 1×10^6^ cfu/mL, 5×10^5^ cfu/mL and 1×10^5^ cfu/mL, respectively. **(C)** The survival rates of *G*. *mellonella* infected with *A*. *fumigati* in 1×10^6^ cfu/mL, 5×10^5^ cfu/mL and 1×10^5^ cfu/mL, respectively.

The survival rates of *G. mellonella* infected with each *A. flavi*, A. *fumigati* or *A. nigri* strain from IPA were compared and results are shown in **Figure 3**. We found that the survival rate of *G. mellonella* larvae infected with *A. flavi*, A. *fumigati* or *A.nigri* strain was dose-dependent. At the 24 h check point, the survival rates of *G. mellonella* larvae infected with *A. flavi* at 1×10^6^ cfu/mL, 5×10^5^ cfu/mL and 1×10^5^ cfu/mL were 5%, 10% and 30%, respectively (*P*<0.05). When compared to 20%, 30% and 100% of *A. nigri-*infected larvae and 45%, 75% and 80% of *A. fumigati*-infected larvae, the survival rates of *A. flavi-*infected larvae are significantly lower at 24 h (*P*<0.05). The similar survival patterns among the three tested *Aspergillus* species were also observed at 36 h and 48 h check points (**Figure 4**).

### Melanization of Galleria mellonellan

**Figure 5:**
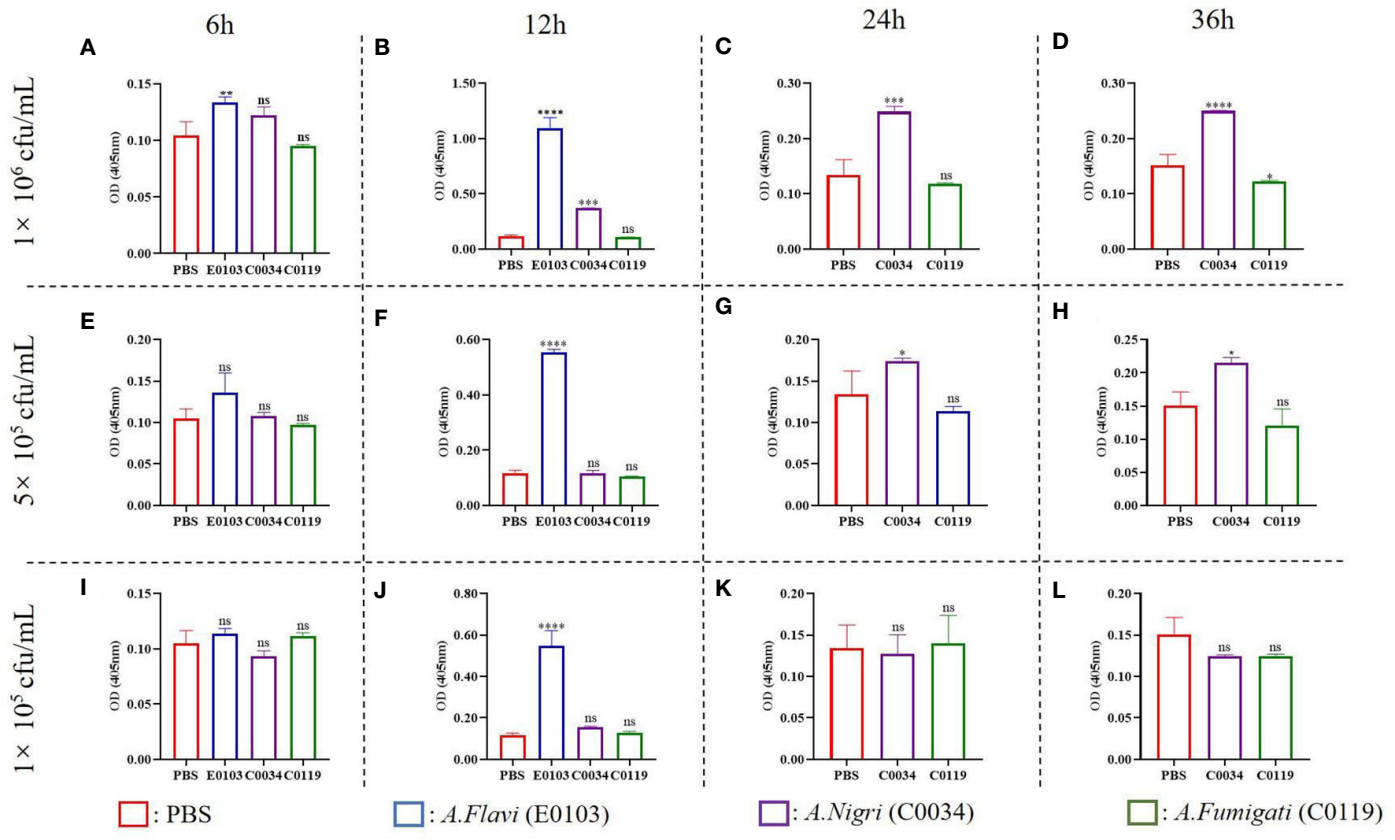
Melanization of G. mellonella larva. **(A)** and **(B)** represent the melanization of *G. mellonella* larva infected with *A. flavi* (E013), *A. fumigati* (C0034) and *A. nigri* (C0119) at 1×10^6^ cfu/mL at 6 h and 12 h, respectively. **(C)** and **(D)** represent the melanization of *G. mellonella* larva infected with *A. fumigati* and *A.nigri* at 1×10^6^ cfu/mL at 24 h and 36 h, respectively. **(E)** and **(F)** represent the melanization of *G. mellonella* larva infected with *A. flavi* (E013), *A. fumigati* (C0034) and *A. nigri* (C0119) at 5×10^5^ cfu/mL at 6 h and 12 h, respectively. **(G)** and **(H)** represent the melanization of *G. mellonella* larva infected with *A.fumigati* and *A.nigri* at 5×10^5^ cfu/mL at 24 h and 36 h, respectively. **(I)** and **(J)** represent the melanization of *G. mellonella* larva infected with *A.flavi * (E013), *A.fumigati* (C0034) and *A.nigri* (C0119) at 1×10^5^ cfu/mL at 6 h and 12 h, respectively. **(K)** and **(L)** represent the melanization of *G. mellonella* larva infected with *A.fumigati* and *A.nigri* at 1×10^5^ cfu/mL at 24 h and 36 h, respectively. “*” present *p* < 0.05, “**” present *p* < 0.01, “***” present *p* < 0.001, “****” present *p* < 0.0001 and “ns” present that there was no significant.

The melanization is a prominent immune response in many insects and arthropods that can be activated by the microbial infection (32). The degrees of melanization in *G. mellonella* larvae infected by *A. flavi*, *A. fumigate* and *A.nigri* isolates from IPA patients were analyzed at different time points as shown in **Figure 5**. At the concentrations of 1×10^6^ cfu/mL and 5×10^5^ cfu/mL infections, the melanization levels of the *G. mellonella* larvae infected by *A. flavi* were significantly higher than the controls at 6 h (*P*<0.05). By 12 h, the melanization levels of *G. mellonella* larvae infected by *A.flavi* with 1×10^6^ cfu/mL, 5×10^5^ cfu/mL and 1×10^5^ cfu/mL were significantly higher than those infected by the same titer concentrations of *A. fumigati* or *A. nigri* (*P*<0.05). This higher melanization level induced by *A. flavi* at 1×10^6^ cfu/mL or 5×10^5^ cfu/mL (i.e., higher than A. *fumigati* and *A. nigri*) persisted at 24 h and 36 h (*P*<0.05). By contrast, the melanization levels of *G. mellonella* larvae infected by *A. fumigati* and *A. nigri* at 5×10^5^ cfu/mL and 1×10^5^ cfu/mL showed no significant difference at 24 h and 36 h (*P >*0.05) (**Figure 5**).

### Histopathology

**Figure 6:**
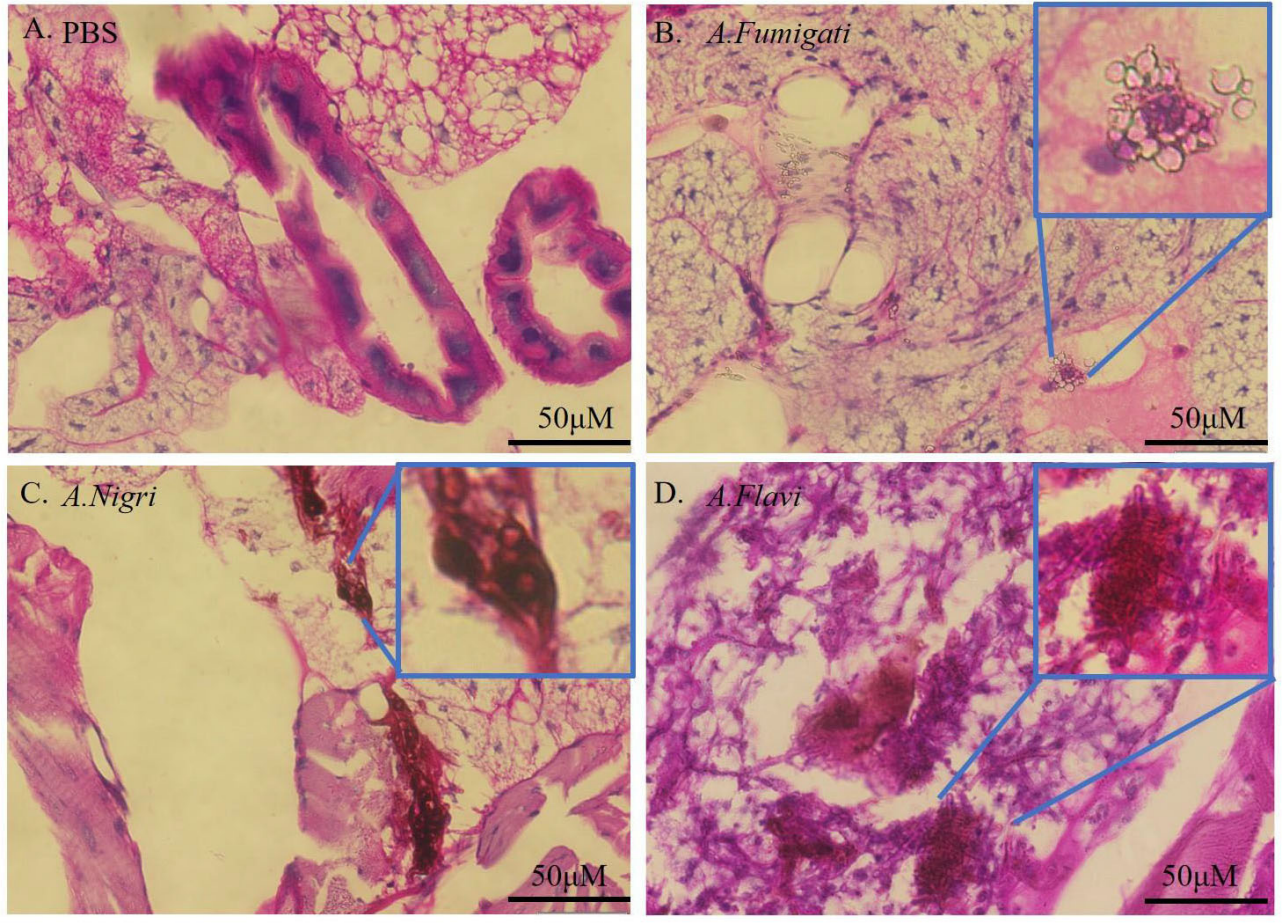
Histopathology of infected *G. mellonella*. **(A-D)** represent the histopathology of *G. mellonella* infected by *A.flavi, A.fumigati* and *A.nigri* at 1×10^6^ cfu/mL at 12 h, respectively.

Histological examination of *G. mellonella* larvae infected by *A. flavi*, *A. fumigate* and *A.nigri* isolates from IPA patients was carried out to study the fungal load in the larval tissue. The results in **Figure 6** show the larval tissues infected by 1×10^6^ cfu/mL of *A. flavi*, A. *fumigati* and *A.nigri* at 12 h post infection. Under PAS staining, along with a large amount of conidia, the hyphae structures of *A. flavi*, A. *fumigati* or *A. nigri* were formed and easily distinguished from *G. mellonella* tissue, particular in *A. flavi*-infected larvae, the clustered hyphae form a hyphal ball in the subcuticular area of larvae. The length of hyphae for *A. flavi*, *A. fumigate* and *A. nigri* all continued to extend as time passed (data not shown).

## Discussion

*Aspergillus* spp. are ubiquitous in the environment, and as saprophytic fungi they will readily contaminate the environment and various food crops, thereby often directly affecting human health (33). In the present study, *CaM* gene sequencing was used to classify the 46 isolates from IA patients in Jining No. 1 People’s Hospital from Shandong province of China. To our surprise, our results showed that *A. flavus* was the most common etiological agent in our collection, followed by *A. fumigatus*, *A. awamori*, *A. terreus*, and *A. tubingensis*, which seems to differ from an earlier study showing that *A. fumigatus* and *A. terreus* were dominant in invasive aspergillosis (34). Indeed, *Aspergillus* strains isolated from our patients covered almost every sections described to be associated with invasive aspergillosis in a early study, including *Fumigati, Flavi, Terrei, Usti, Nigri and Nidulantes* (35).

IPA is the most severe type of pulmonary aspergillosis and evidence showed that immunocompromised hosts are particularly prone to IPA and cytokines play a pivotal role in the infection and progress (36). In the present study, we found that CRP levels and counts of total leukocytes, neutrophils, and monocytes in IPA patients were significantly higher than in patients in complete health (*P*<0.05), but lymphocytes were lower. CRP is a classical marker of inflammation and can be a predictor for the invasive aspergillosis (37). The elevation of CPR levels is similar to other reports in which inflammatory biomarkers were significantly increased in the patients with proven aspergillosis (38).

*Aspergillus* section *Flavi* includes 33 species and most of them are natural aflatoxin producers. In the present study, 25 clinical isolates (54.3%) were identified as *A. flavus*. As others have noted, *A. flavus* is the second leading cause of invasive aspergillosis after *A. fumigatus* (36). The cause of the observed dominance of *Aspergillus* section *Flavi* in our collection is not understood. Given the fact that our hospital serves patients mostly working on farms or elsewhere in the agriculture industry, they may be infected through contact with *A. flavus*-rich living environments such as the air and local common crops (wheat and peanuts). It has also been reported that section *Flavi* is often isolated from superficial infections such as keratitis, cutaneous infections and osteomyelitis after reported trauma (38). To our knowledge, the pathogenicity and antifungal susceptibility of *A. flavus* are not well studied compared to *A. fumigatus*. The antifungal susceptibility test in this study indicated that all *A. flavus* strains were susceptible to Terbinafine, Voriconazole and Itraconazole. A recent study has also shown that *A. flavus* strains were more susceptible to Voriconazole and Itraconazole than *A. fumigatus* (37). The absence of azole-resistant strains together with the greater prevalence of *A. flavus* in our local area may afford us some additional time to treat invasive aspergillosis by using azole drugs, but we should never lose sight of the fact that this time is borrowed, and is limited. However, we observed that 5 A*. flavus* isolates showed MICs > 8 µg/mL for Micafungin and >16 µg/mL for Amphotericin B. The differences in antifungal susceptibilities among these clinical isolates is not explored here, but may be related to genetic and specific treatment factors (39).

*Fumigati* section has been reclassified recently and at present contains 25 species (40). *A. fumigatus* is the most common human pathogen (36). In the present study, antifungal susceptibility tests indicated that all 9 A*. fumigatus* strains were still susceptible to Voriconazole and Itraconazole. These results are consistent with a previous study in which *A. fumigatus* strains had relatively low MICs to triazole drugs (41). *Nigri* section contains eight different species, among which *A. acidus*, *A. awamori*, *A. brasiliensis*, *A. niger* and *A. tubingensis* share morphological characteristics so similar (42) that a molecular technology is absolutely required to differentiate them at the species level. In the present study, *A. awamori* and *A. tubingensis* accounted for 19.6% and 2.2% in our collection. Our data also showed that *A. tubingensis* was the most common species among patients with type 2 diabetes mellitus (5). The antifungal susceptibility tests showed that *A. awamori* and *A. tubingensis* strains were more susceptible to Micafungin and Terbinafine, but resistant to Amphotericin B, Voriconazole, Itraconazole and Fluconazole. The resistances to Voriconazole and Itraconazole were stronger in *A. tubingensis* strains, suggesting a higher dose or alternative drug is required for effective treatment (5).

Our isolated *A. flavus* strain also presented a higher virulence than *A. fumigatus* and *A. niger* in the *G. mellonella* larvae model (43). As a species that produces a most popular aflatoxin (44), *A. flaus*-infected larvae showed a lower survival rate and more fungal structures in affected subcuticular areas. At the same time, a stronger host response (melanization level) was observed in *A. flavus*-infected larvae. However, whether this mycotoxin contributes to its virulence is unknown.

In summary, *Aspergillus flavus* was the dominant clinical etiological agent. The CRP level, the total numbers of leukocytes, neutrophilic granulocytes, and monocytes of IPA are significantly higher than those in healthy hosts – even though the reduced lymphocyte levels remain somewhat puzzling. We found that 91%, 31%, 14%, 14% of *Aspergillus* strains from IPA patients were resistant to Fluconazole, Micafungin, Amphotericin B and Terbinafine, respectively. The survival rates of larvae infected with *A. flavus* were much lower than those infected by *A. niger* or *A. fumigatus* (*P* < 0.05). Although the antimicrobial susceptibility and virulence data may not actually reflect *in vivo* effects, we believe this study provides some guidance for treatment choices in patients with IPA.

## Data availability statement

The datasets presented in this study can be found in online repositories. The names of the repository/repositories and accession number(s) can be found in the article/**Supplementary Material**.

## Author contributions

All authors contributed to the article and approved the submitted version. BC performed the experiments and wrote the manuscript. GQ, ZY and NZ: Performed the experiments. DL Analyzed the data. DS: Wrote and revised the manuscript.

## References

[1] Gianni C Romano C. Clinical and histological aspects of toenail onychomycosis caused by *aspergillus* spp.: 34 cases treated with weekly intermittent terbinafine. Dermatology (2004) 209:104–10. doi: 10.1159/000079593 15316163

[2] Balajee SA Houbraken J Verweij PE Hong SB Yaghuchi T Varga J. *Aspergillus* species identification in the clinical setting. Stud Mycol (2007) 59:39–46. doi: 10.3114/sim.2007.59.05 18490954 PMC2275201

[3] Visagie CM Houbraken J. Updating the taxonomy of *aspergillus* in south africa. Stud Mycol (2020) 95:253–92. doi: 10.1016/j.simyco.2020.02.003 PMC7426233 32855741

[4] Shcab C Yclb D Cjlb D Pskb D Hywb D Lthb D. Invasive mold infections in acute leukemia patients undergoing allogeneic hematopoietic stem cell transplantation. J Microbiol Immunol Infect (2019) 52:973–82. doi: 10.1016/j.jmii.2018.09.006 30322746

[5] Frias-De-Leon MG Rosas-de Paz E Arenas R Atoche C Duarte-Escalante E Molina de Soschin D. Identification of *aspergillus tubingensis* in a primary skin infection. J Mycol Med (2018) 28:274–8. doi: 10.1016/j.mycmed.2018.02.013 29551443

[6] Jing R Yang WH Xiao M Li Y Hsueh PR. Species identification and antifungal susceptibility testing of aspergillus strains isolated from patients with otomycosis in northern china. J Microbiol Immunol Infect (2021) 55:282–90. doi: 10.1016/j.jmii.2021.03.011 33839057

[7] Donnelly JP Chen SC Kauffman CA Steinbach WJ Baddley JW Verweij PE. Revision and update of the consensus definitions of invasive fungal disease from the european organization for research and treatment of cancer and the mycoses study group education and research consortium. Clin Infect Dis (2019) 71(6):1367–1376. doi: 10.1093/cid/ciz1008 PMC7486838 31802125

[8] Durieux MF Melloul L Jemel S Roisin L Marie-Laure D Guillot J. *Galleria mellonella* as a screening tool to study virulence factors of aspergillus fumigatus. Virulence (2021) 12:818–34. doi: 10.1080/21505594.2021.1893945 PMC7946008 33682618

[9] Naaraayan A Kavian R Lederman J Basak P Jesmajian S. Invasive pulmonary aspergillosis - case report and review of literature. J Community Hosp Intern Med Perspect (2015) 5(1):26322. doi: 10.3402/jchimp.v5.26322 25656673 PMC4318821

[10] Frisvad JC Larsen TO. Extrolites of aspergillus fumigatus and other pathogenic species in aspergillus section *fumigati* . Front Microbiol (2016) 6:1485. doi: 10.3389/fmicb.2015.01485 26779142 PMC4703822

[11] Greiner K PerOh D Weig A Rambold G. *Phialosimplex salinarum*, a new species of eurotiomycetes from a hypersaline habitat. Ima Fungus (2014) 5:161–72. doi: 10.5598/imafungus.2014.05.02.01 PMC4329318 25734026

[12] Subcommittee on Antifungal Susceptibility Testing of the ESCMID European Committee for Antimicrobial Susceptibility Testing. EUCAST technical note on the method for the determination of broth dilution minimum inhibitory concentrations of antifungal agents for conidia-forming moulds. Clin Microbiol Infect (2008) 14(10):982–4. doi: 10.1111/j.1469-0691.2008.02086.x 18828858

[13] Sigler L Sutton DA Gibas CFC Summerbell RC Noel RK Iwen PC. *Phialosimplex*, a new anamorphic genus associated with infections in dogs and having phylogenetic affinity to the. Trichocomaceae Med Mycol (2010) 48:335–45. doi: 10.3109/13693780903225805 20141373

[14] Howard SJ. Multi-resistant aspergillosis due to cryptic species. Mycopathologia (2014) 178:435–9. doi: 10.1007/s11046-014-9774-0 24969615

[15] Visagie CM Varga J Houbraken J Meijer M Kocsubé S Yilmaz N. Ochratoxin production and taxonomy of the *yellow aspergilli* (aspergillus section circumdati). Stud Mycol (2014) 78:1–61. doi: 10.1016/j.simyco.2014.07.001 25492980 PMC4255584

[16] Balajee SA Marr KA. Phenotypic and genotypic identification of human pathogenic aspergill. Future Microbiol (2006) 1:435–45. doi: 10.2217/17460913.1.4.435 17661634

[17] Alcazar-Fuoli L Mellado E Alastruey-Izquierdo A Cuenca-Estrella M Rodriguez-Tudela JL. Aspergillus section *fumigati*: antifungal susceptibility patterns and sequence-based identification. Antimicrob Agents Chemother (2008) 52:1244–51. doi: 10.1128/AAC.00942-07 PMC2292508 18212093

[18] Balajee SA Weaver M Imhof A Gribskov J Marr KA. *Aspergillus fumigatus* variant with decreased susceptibility to multiple antifungals. Antimicrob Agents Chemother (2004) 48:1197–203. doi: 10.1128/AAC.48.4.1197-1203.2004 PMC375298 15047520

[19] Samson RA Visagie CM Houbraken J Hong SB Hubka V Klaassen CH. Phylogeny, identification and nomenclature of the genus aspergillus. Stud Mycol (2014) 78:141–73. doi: 10.1016/j.simyco.2014.07.004 PMC4260807 25492982

[20] Sanchez EKC Almaguer CM Duarte EE Rojas FT Frías-De-León MG Reyes-Montes MDR. Aspergillus phylogenetic identification, diversity, and richness of aspergillus from homes in havana, cuba. Microorganism (2021) 9:115. doi: 10.3390/microorganisms9010115 PMC7825327 33418970

[21] Matsuzawa THY. *Aspergillus takadae*, a novel heterothallic species of *aspergillus* section fumigati isolated from soil in china. Mycoscience (2019) 60:354–60. doi: 10.1016/j.myc.2019.07.003

[22] Hubka V Barrs V Dudova Z Sklenar F Kubátová A Matsuzawa T. Unravelling species boundaries in the aspergillus viridinutans complex (section fumigati): Opportunistic human and animal pathogens capable of interspecific hybridization. Persoonia (2018) 41:142–74. doi: 10.3767/persoonia.2018.41.08 PMC6344812 30728603

[23] Zhang YS Ding G Sun BD Zhou YG Zhao GZ Chen AJ. Phylogeny of *aspergillus* section terrei with two new records from the china general microbiological culture collection centre. Phytotaxa (2018) 382:275–87. doi: 10.11646/phytotaxa.382.3.4

[24] Howard SJ Harrison E Bowyer P Varga J Denning DW. Cryptic species and azole resistance in the aspergillus niger complex. Antimicrob Agents Chemother (2011) 55:4802–9. doi: 10.1128/AAC.00304-11 PMC3186969 21768508

[25] Kaskatepe B Aslan Erdem S Ozturk S Safi Oz Z Subasi E Koyuncu M. Antifungal and anti-virulent activity of *origanum majorana l.* essential oil on *Candida albicans* and *in vivo* toxicity in the *galleria mellonella* larval model. Molecules (2022) 27:663. doi: 10.3390/molecules27030663 35163928 PMC8838586

[26] Garcia-Bustos V Ruiz-Saurí A Ruiz-Gaitán A Sigona-Giangreco IA Cabañero-Navalon MD. Characterization of the differential pathogenicity of *Candida auris* in a *galleria mellonella* infection model. Microbiol Spectr (2021) 9:e0001321. doi: 10.1128/Spectrum.00013-21 34106570 PMC8552516

[27] Wrońska AK Boguś MI. Harman and norharman, metabolites of the entomopathogenic fungus *Conidiobolus coronatus* (Entomophthorales), affect the serotonin levels and phagocytic activity of hemocytes, insect immunocompetent cells, in *galleria mellonella* (Lepidoptera). Cell Biosci (2019) . 9:29. doi: 10.1186/s13578-019-0291-1 30962871 PMC6434831

[28] Vertyporokh L Hulas-Stasiak M Wojda I. Host-pathogen interaction after infection of *galleria mellonella* with the filamentous fungus *beauveria bassiana* . Insect Sci (2020) 27:1079–89. doi: 10.1111/1744-7917.12706 PMC7497211 31245909

[29] Mukherjee K Vilcinskas A. The entomopathogenic fungus metarhizium robertsii communicates with the insect host *galleria mellonella* during infection. Virulence (2017) 9:402–13. doi: 10.1080/21505594.2017.140519 PMC5955202 29166834

[30] Tsai C Loh JMS Proft T. *Galleria mellonella* infection models for the study of bacterial diseases and for antimicrobial drug testing. Virulence (2016) 7:214–29. doi: 10.1080/21505594.2015.1135289 PMC4871635 26730990

[31] Singkum P Suwanmanee S Pumeesat P Luplertlop N. A powerful *in vivo* alternative model in scientific research: *Galleria mellonella* . Acta Microbiol Immunol Hung (2019) 66:31–55. doi: 10.1556/030.66.2019.001 30816806

[32] Walsh TJ Anaissie EJ Denning DW Herbrecht R Kontoyiannis DP Marr KA. Treatment of aspergillosis: clinical practice guidelines of the infectious diseases society of america. Clin Infect Dis (2008) 46:327–60. doi: 10.1086/525258 18177225

[33] Tang H. Regulation and function of the melanization reaction in drosophila. Fly (Austin) (2009) 3:105–11. doi: 10.4161/fly.3.1.7747 19164947

[34] Norlia M Jinap S Nor-Khaizura MAR Radu S Samsudin NIP Azri FA. *Aspergillus* section flavi and aflatoxins: occurrence, detection and identification in raw peanuts and peanut-based products along the supply chain. Front Microbiol (2019) 10:2602. doi: 10.3389/fmicb.2019.02602 31824445 PMC6886384

[35] Mayr A Lass-FlRl C. Epidemiology and antifungal resistance in invasive *aspergillosis* according to primary disease - review of the literature. Eur J Med Res (2011) 16(4):153–7. doi: 10.1186/2047-783x-16-4-153 PMC3352071 21486729

[36] Samson RA Hong SB Frisvad JC. Old and new concepts of species differentiation in. aspergillus Med Mycol (2006) 44:S133–48. doi: 10.1080/13693780600913224 30408897

[37] Donnelly JP Chen SC Kauffffman CA Steinbach WJ Baddley JW Verweij PE. Revision and update of the consensus definitions of invasive fungal disease from the european organization for research and treatment of cancer and the mycoses study group education and research consortium. Clin Infect Dis (2020) 16:153–157. doi: 10.1093/cid/ciz1008 PMC7486838 31802125

[38] Ullmann AJ Aguado JM Arikan-Akdagli S Denning DW Groll AH Lagrou K. Diagnosis and management of *Aspergillus* diseases: executive summary of the 2017 ESCMID-ECMM-ERS guideline. Clin Microbiol Infect (2018) 44:S133–S148. doi: 10.1016/j.cmi.2018.01.002 29544767

[39] Chai L Netea MG Teerenstra S Earnest A Vonk AG Schlamm HT. Early proinflammatory cytokines and c-reactive protein trends as predictors of outcome in invasive aspergillosis. J Infect Dis (2010) e1–e38. doi: 10.1086/656527 20879853

[40] Frisvad JC Hubka V Ezekiel CN Hong SB Nováková A Chen AJ. Taxonomy of *aspergillus* section flavi and their production of aflatoxins, ochratoxins and other mycotoxins. Stud Mycol (2019) 93:1–63. doi: 10.1016/j.simyco.2018.06.001 30108412 PMC6080641

[41] Hedayati MT Pasqualotto AC Warn PA Bowyer P Denning DW. *Aspergillus flavus*: human pathogen, allergen and mycotoxin producer. Microbiology (2007) 153:1677–92. doi: 10.1099/mic.0.2007/007641-0 17526826

[42] Tsang CC Tang JYM Ye H Xing F Lo SKF Xiao C. Rare/cryptic *aspergillus* species infections and importance of antifungal susceptibility testing. Mycoses Dis (2020) 63:1283–98. doi: 10.1111/myc.13158 32918365

[43] Mohamadnia A Salehi Z Namvar Z Tabarsi P Pourabdollah-Toutkaboni M Rezaie S. Molecular identification, phylogenetic analysis and antifungal susceptibility patterns of aspergillusnidulans complex and aspergillusterreus complex isolated from clinical specimens. J Mycol Med (2020) 30:101004. doi: 10.1016/j.mycmed.2020.101004 32534826

[44] Balajee SA Gribskov JL Hanley E Nickle D Marr KA. *Aspergilluslentulus* sp. nov., a new sibling species of *A. fumigatus* . Eukaryot Cell (2005) 4:625–32. doi: 10.1128/EC.4.3.625-632.2005 PMC1087803 15755924

