# Supplementary material for: Virulence capacity of different Aspergillus species from invasive pulmonary aspergillosis
Source: Front Immunol. 2023 Mar 29;14:1155184. doi: 10.3389/fimmu.2023.1155184 (PMC10090689; doi:10.3389/fimmu.2023.1155184)
Supplement: Supplementary file 1 [file Table_1.docx]

**Table S1.** Clinical *Aspergillus* isolates with genbank numbers.

| **Isolates** | **Genbank no.** | **Sex** | **Age** |
| --- | --- | --- | --- |
|  |  |  |  |
| CCJNMMM-C-0001 | MZ207999 | Female | 37 years |
| CCJNMMM-C-0002 | MZ208000 | Female | 37 years |
| CCJNMMM-C-0003 | MZ208001 | Male | 85 years |
| CCJNMMM-C-0032 | MZ208002 | Male | 51 years |
| CCJNMMM-E-0114 | MZ208003 | Male | 76 years |
| CCJNMMM-E-0136-1 | MZ208004 | Male | 58 years |
| CCJNMMM-E-0136-2 | MZ208005 | Male | 58 years |
| CCJNMMM-C-0007 | MZ208006 | Female | 86 years |
| CCJNMMM-C-0016 | MZ208007 | Female | 53 years |
| CCJNMMM-C-0018 | MZ208008 | Female | 83 years |
| CCJNMMM-C-0020 | MZ208009 | Female | 82 years |
| CCJNMMM-C-0025 | MZ208010 | Female | 2 months |
| CCJNMMM-C-0026 | MZ208011 | Male | 67 years |
| CCJNMMM-C-0030 | MZ208012 | Female | 84 years |
| CCJNMMM-C-0034 | MZ208013 | Male | 93 years |
| CCJNMMM-C-0036 | MZ208014 | Male | 30 years |
| CCJNMMM-C-0037 | MZ208015 | Male | 42 years |
| CCJNMMM-C-0038 | MZ208016 | Female | 66 years |
| CCJNMMM-C-0054 | MZ208017 | Male | 75 years |
| CCJNMMM-C-0074 | MZ208018 | Female | 64 years |
| CCJNMMM-C-0119 | MZ208019 | Female | 75 years |
| CCJNMMM-C-0121 | MZ208020 | Male | 68 years |
| CCJNMMM-C-0122 | MZ208021 | Male | 9 months |
| CCJNMMM-C-0125 | MZ208022 | Male | 57 years |
| CCJNMMM-C-0139 | MZ208023 | Male | 82 years |
| CCJNMMM-C-0156 | MZ208024 | Male | 66 years |
| CCJNMMM-C-0178 | MZ208025 | Male | 72 years |
| CCJNMMM-E-0104 | MZ208026 | Female | 4 years |
| CCJNMMM-E-0108 | MZ208027 | Female | 15 years |
| CCJNMMM-E-0112 | MZ208028 | Female | 42 years |
| CCJNMMM-E-0115 | MZ208029 | Female | 38 years |
| CCJNMMM-E-0116 | MZ208030 | Female | 4 months |
| CCJNMMM-E-0117 | MZ208031 | Female | 88 years |
| CCJNMMM-E-0138 | MZ208032 | Female | 72 years |
| CCJNMMM-E-0163 | MZ208033 | Female | 57 years |
| CCJNMMM-E-0164 | MZ208034 | Female | 36 years |
| CCJNMMM-E-0181 | MZ208035 | Female | 17 years |
| CCJNMMM-C-0011 | MZ208036 | Female | 85 years |
| CCJNMMM-C-0031 | MZ208037 | Female | 73 years |
| CCJNMMM-C-0158 | MZ208038 | Female | 71 years |
| CCJNMMM-E-0107 | MZ208039 | Male | 82 years |
| CCJNMMM-E-0110 | MZ208040 | Female | 50 years |
| CCJNMMM-C-0133 | MZ208041 | Male | 91 years |
| CCJNMMM-E-0103 | MZ208042 | Female | 73 years |
| CCJNMMM-E-0118 | MZ208043 | Male | 82 years |
| CCJNMMM-E-0100 | MZ208044 | Male | 54 years |
